# Supplementary material for: Missing checkerboards? An absence of competitive signal in Alnus-associated ectomycorrhizal fungal communities
Source: PeerJ. 2014 Dec 16;2:e686. doi: 10.7717/peerj.686 (PMC4273934; doi:10.7717/peerj.686)

Figure S1. Root density in the three research plots. Plot 2 = 268 stems/ha, Plot 4 = 1557, Plot 8 = 3559. Values based on ten replicate 1 cubic liter soil cores taken throughout each plot on 31 May, 2013. No significant differences were found (one-way ANOVA: F = 0.14, DF = 2,27, P = 0.869).

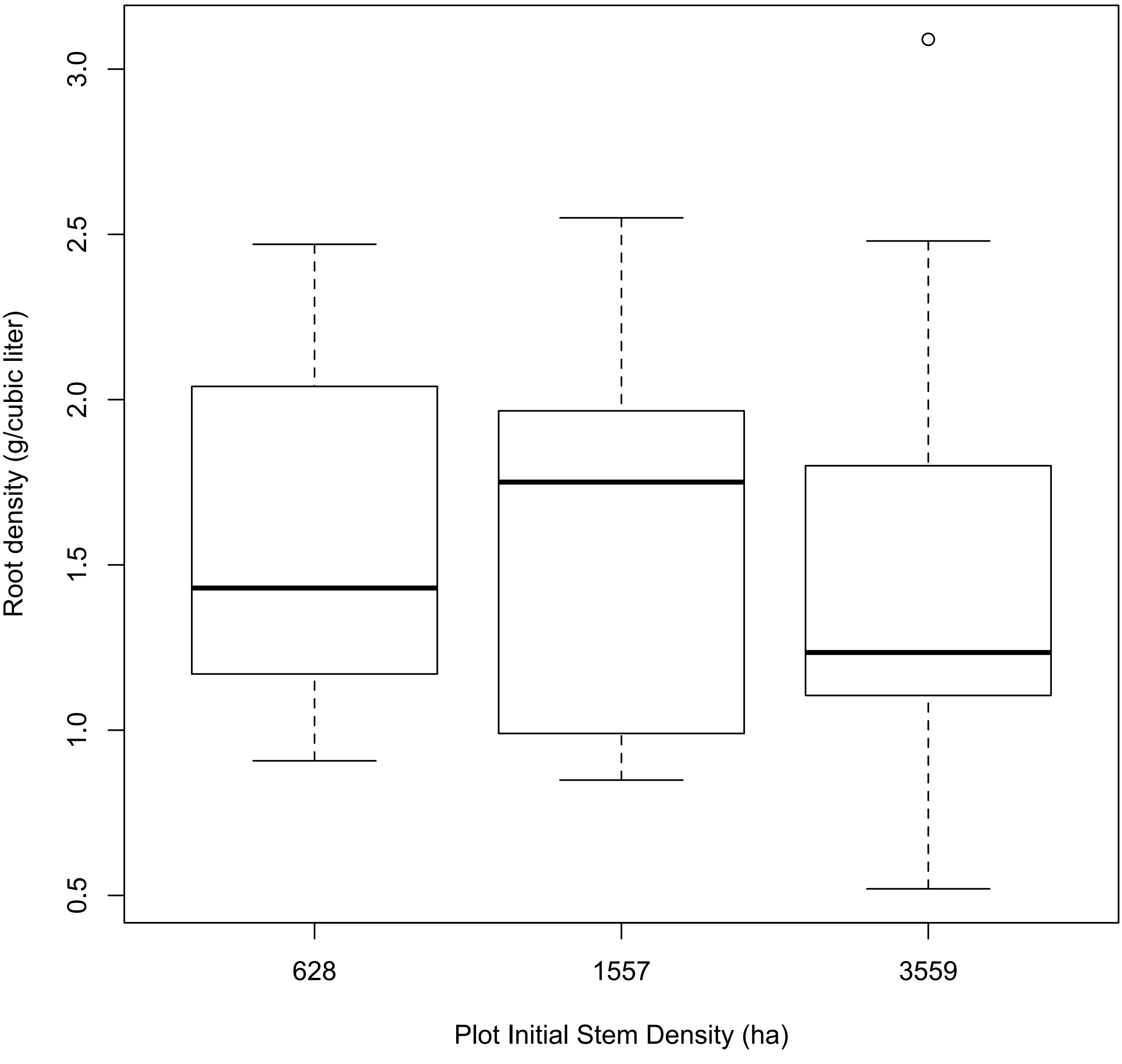

Supplement: Figure S1 — Plot 2 = 268 stems/ha, Plot 4 = 1,557, Plot 8 = 3,559. Values based on ten replicate 1 cubic liter soil cores taken throughout each plot on 31 May, 2013. No significant differences were found (one-way ANOVA: F = 0.14, DF = 2, 27, P = 0.869). [file peerj-02-686-s001.pdf]
