# Supplementary figures and images for: Missing checkerboards? An absence of competitive signal in Alnus-associated ectomycorrhizal fungal communities
Source: PeerJ. 2014 Dec 16;2:e686. doi: 10.7717/peerj.686 (PMC4273934; doi:10.7717/peerj.686)

Figure S2. Mantel correlogram of Plot2 Alnus dataset.

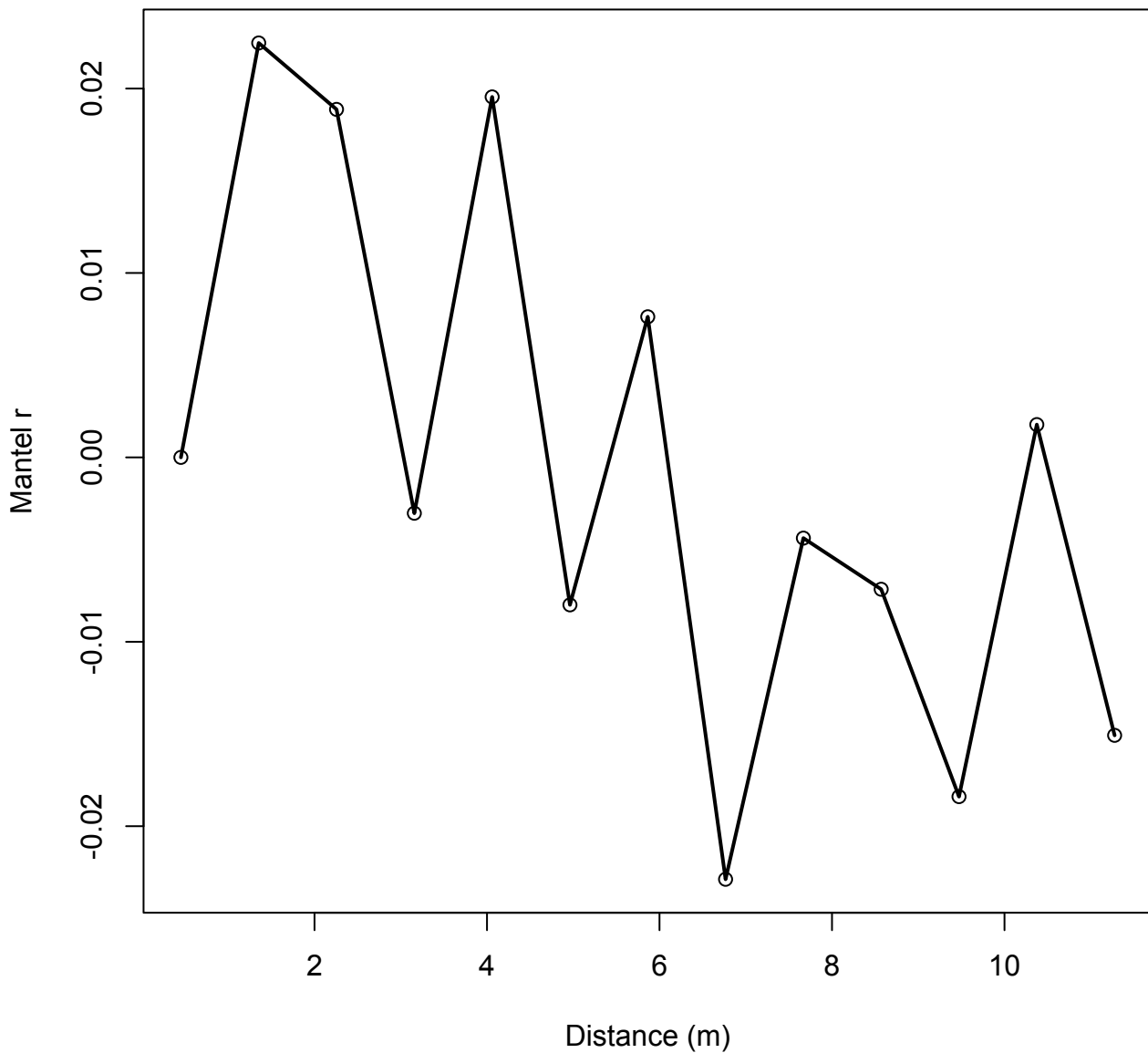

Supplement: Figure S2 [file peerj-02-686-s002.pdf]

Figure S3. Mantel correlogram of Plot8 Alnus dataset.

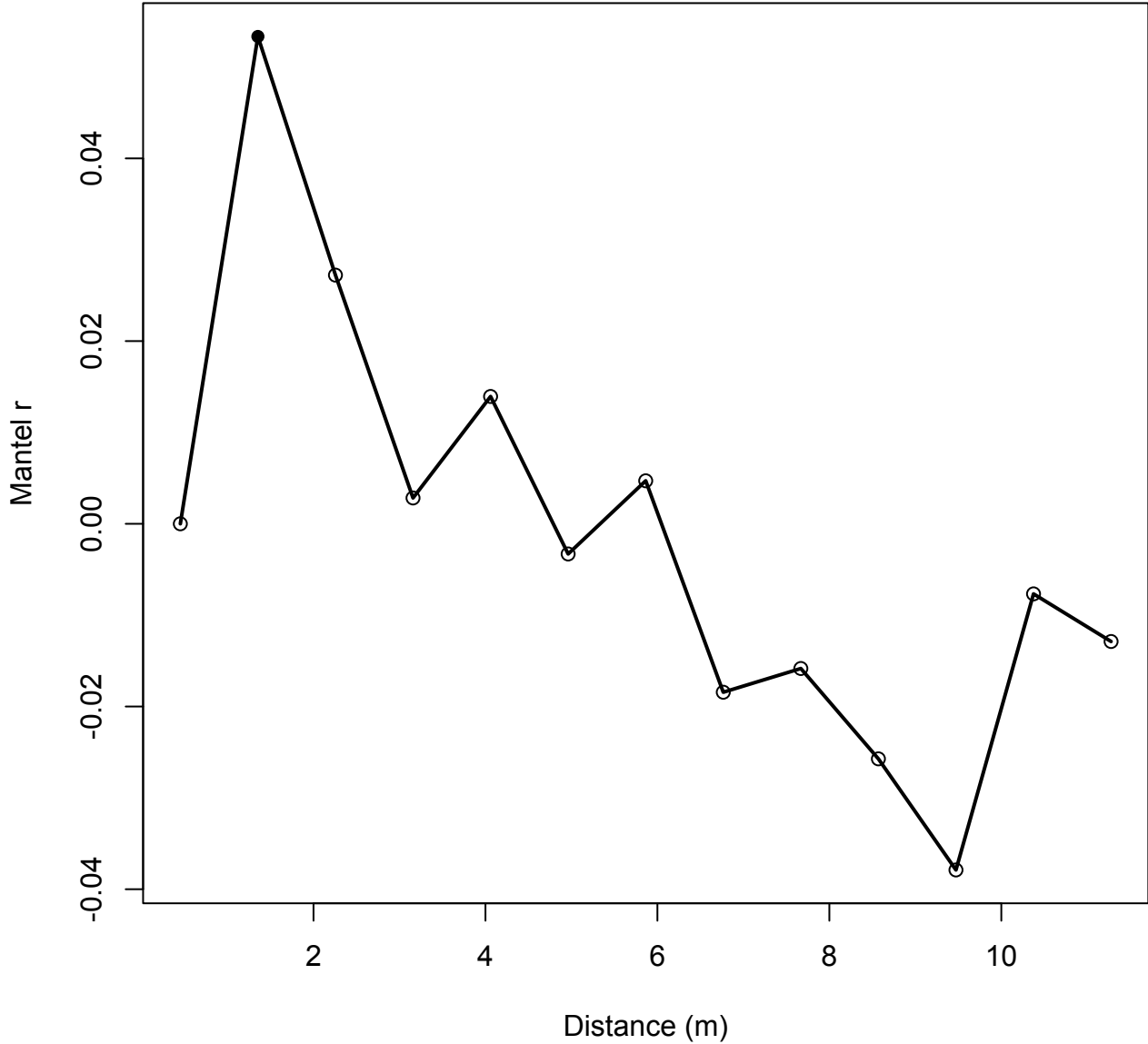

Supplement: Figure S3 [file peerj-02-686-s003.pdf]
